# The association of smoking and alcohol in colorectal cancer in black patients – Case-control study

**DOI:** 10.4102/jphia.v15i1.532

**Published:** 2024-10-04

**Authors:** Mpho K. Kgomo, Ratidzo L. Zingoni, Piet J. Becker

**Affiliations:** 1 Department of Gastroenterology Medicine, Faculty of Health Sciences, University of Pretoria, Pretoria, South Africa; 2 Department of Internal Medicine, Faculty of Health Sciences, University of Pretoria, Pretoria, South Africa; 3 Department of Biostatistics, Faculty of Health Sciences, University of Pretoria, Pretoria, South Africa

**Keywords:** large bowel, neoplasm, tobacco, alcohol, African

## Abstract

**Background:**

Studies have focused on smoking and alcohol as risk factors for colorectal cancer (CRC). Caucasians and other populations have been studied worldwide, and both smoking and alcohol have been validated as causes of CRC. However, there are limited data on the black population; studies that have been performed in Africa have not specifically focused on these two risk factors but rather in combination with other risks.

**Aim:**

To determine how smoking and alcohol affect the incidence of CRC in the African black population.

**Setting:**

Steve Biko Academic Hospital’s gastrointestinal clinic.

**Methods:**

Subjects used for the study included black African patients above 18 years who had undergone a colonoscopy for suspected CRC between 2016 and 2018. Cases used were confirmed CRC on histology; controls were negative on histology. A minimum of 68 cases and 136 controls were needed for this study according to sample calculation. Hundred and ten cases and 220 controls were obtained in the final analysis. Data were collected between June 2019 and March 2020.

**Results:**

Smoking (odds ratio [OR] = 1.795, *p* = 0.049) was a significant risk factor for CRC among black patients who presented at the gastrointestinal clinic. Age > 50 years (OR = 3.742, *p* < 0.001), family history (OR = 12.457, *p* < 0.001), and the combination of smoking and alcohol (OR = 5.927, *p* = 0.008) were significant risk factors. Interestingly, alcohol alone was protective (OR = 0.205, *p* < 0.001).

**Conclusion:**

Both smoking and a combination of alcohol and smoking are significant risk factors in the development of CRC in the black African population.

**Contribution:**

Smoking, as in most population groups, is a risk factor for CRC. The observed protective role of alcohol needs to be confirmed in larger studies representing the African population.

## Introduction

### Literature overview and motivation

Colorectal cancer (CRC) is the third most common cancer and the fourth most common cause of cancer-related deaths worldwide.^1^ The GLOBOCAN project, which aims to provide contemporary estimates of the incidence, mortality rate and prevalence of major types of cancer at the national level for 184 countries worldwide, estimates that 1.2 million cases of CRC and 600 000 deaths occur each year.^2^ Despite major improvements in early diagnosis and therapy, the 5-year relative survival rate is still < 65%, even in highly developed countries.^2^

Lifestyle factors associated with CRC risk include smoking, alcohol intake, high red meat and processed meat consumption, high-fat and high-protein diet intake, physical inactivity and being overweight. According to the National Institute for Clinical Excellence, about three-quarters of CRC cases are associated with the population’s lifestyle.^2,3^ There have been a number of studies in Africa on the demographic and lifestyle characteristics associated with CRC. However, studies are lacking on how the incidence of smoking and alcohol specifically contribute to the development of CRC in black Africans. This is important, as risk and survival trends and how they lead to incidence need monitoring in order to provide a comprehensive picture of the disease.^4^

This literature review focuses on what has been found concerning CRC in black African populations, what is already known about smoking and alcohol, and how these contribute to the development of CRC. The databases UpToDate and PubMed were used, and the searched groups of keywords included ‘colorectal cancer, epidemiology, Africa’, ‘colorectal cancer, epidemiology, sub-Saharan Africa’, ‘colorectal cancer, alcohol, Africa’ and ‘colorectal cancer, smoking, Africa’.

### Presentation of colorectal cancer

Patients with CRC typically present in three ways: suspicious symptoms and/or signs (such as haematochezia or melena, abdominal pain, unexplained iron deficiency anaemia and changes in bowel habits); asymptomatic individuals discovered by routine screening; or, finally, as emergency presentations with obstruction, peritonitis or, rarely, acute gastrointestinal (GIT) bleeding.^5^

Although the increase in CRC screening has led to more cases being diagnosed at an asymptomatic stage, most cases are diagnosed after the onset of symptoms. Indeed, the commonest clinical symptoms presented in a study performed at the Komfo Anokye Teaching Hospital, Ghana, were weight loss (44.80%), bleeding per rectum (39.82%) and abdominal pain (38.91%).^6^ Symptoms of CRC are typically because of growth of the tumour into the lumen or adjacent structures; as a result, symptomatic presentation usually reflects relatively advanced CRC.^5^

To elaborate, the Ghana study^6^ aimed to determine the clinical features and histopathological patterns of CRC nationally, using 221 CRC cases collected by retrospective review of patient records. The investigators found that 7.24% of patients had a family history of cancer. Lifestyle characteristics of the studied participants also showed a 24.89% prevalence of comorbidities, with 8.14% diabetes, 19.91% hypertension and 4.07% with both. It was also found that 9.50% of the studied participants were alcohol users, 4.98% were smokers and 4.07% were both alcohol users and smokers. The majority of patients (40.27%) were identified as being in late-stage CRC development (TNM stage III). The overall crude annual incidence was 4.62 per 100 000 people, demonstrating an incidence of CRC similar to that of most African countries. Lesions were predominantly rectal; adenocarcinoma (68.33%) was the most common histopathological type. Of concern was the high incidence among younger people and delayed disease presentation at advanced stages.^6^ Notably, the study also found a higher prevalence of CRC in females. This could be attributed to the high prevalence of obesity among females in Ghana and/or the high hospital attendance by females compared to males in this local setting, as females are more conscious of their health and report more speedily to the hospital.^6^

In another study, based in Zimbabwe,^7^ data on cases of CRC recorded by the Zimbabwe National Cancer Registry between 2003 and 2012 were collected. The demographic and pathological characteristics were compared according to ethnicity and age. Signet ring cell carcinomas were more common among black Africans than in caucasians (4% vs. 1%, *p* = 0.027).

Similarly, Baroudi et al.^8^ investigated the impact of lifestyle factors and nutrient intake on GIT cancer occurrence in the Tunisian population. They found that the consumption of vegetables, fruit, fish and coffee was protective, while tobacco, alcohol and tea posed an increased risk.^8^

Finally, living in low socioeconomic status areas was also shown to be associated with later stage at diagnosis and, therefore, lower resection rates. This was demonstrated in a study performed in Durban, South Africa, which compared patients diagnosed with CRC in the state and private sector hospitals over a 12-month period (January 2009 – December 2009). More advanced disease at diagnosis was found among state patients. The resection rate of 81% for the private sector resembled that of 79% – 89% reported worldwide, while that in the state was 61%, reflecting a tendency to present with advanced disease.^9^

### Smoking and colorectal cancer

Historically, literature states that with respect to the GIT tract, both oesophageal and gastric cancers have been strongly associated with tobacco. The smoking–CRC link, however, remains unclear.

Botteri et al.^10^ performed a literature review investigating the relationship between smoking and CRC. They found several large cohort studies linking smoking with CRC and the risk of colorectal polyps. However, most failed to detect a significant relationship between smoking and CRC. They theorised that this could be explained by different study designs, population characteristics and the heterogeneous treatment of the most likely confounders, such as diet, alcohol, physical activity and body mass index, which had been the basis for the literature review. A statistically significant increasing dose relationship was found for a number of pack-years and cigarettes per day, but only after 30 years of smoking. Seventeen cohort studies were included in the mortality analysis. The pooled risk estimate (forever vs. never smokers) was 1.25 (95% confidence interval [CI]: 1.14–1.37), and smoking was associated with an absolute risk increase of 6.0 deaths per 100 000 person-years (95% CI: 4.2–7.6). For both incidence and mortality, the association was stronger for rectal cancer than for colon cancer. It was concluded that cigarette smoking was significantly associated with CRC incidence and mortality.

Furthermore, studies conducted at the Minia Oncology Centre in Egypt aimed to determine the relation between dietary and lifestyle factors and CRC development, using controls without the disease for comparison. Both active and passive smoking were found to be significantly more frequent among CRC patients (34% vs. 18.7% and 24.7% vs. 5.3%, respectively).^11^ Similarly, findings reported by Limsui et al.,^12^ who conducted a prospective study on the relation between cigarette smoking and CRC risk, found that 34% of those who developed CRC were smokers. Finally, Abdulbari et al.^13^ also carried out a case-control study on lifestyle habits and CRC risk in Qatar, and found that 26.7% of cases and 17% of controls were smokers.

### Alcohol and colorectal cancer

To determine whether there was a link between alcohol and CRC, Giovannucci et al.^14^ found that alcohol intake may enhance the risk of developing distal CRCs, although the evidence was not entirely consistent. They noted that the influence of alcohol may be particularly strong when combined with a diet low in methionine and folate, suggesting that the effect of alcohol may involve antagonism of methyl group metabolism. The combined effect of these dietary factors, as well as modifiable nondietary factors, such as cigarette smoking, suggested that the majority of colon cancer cases are preventable.^14^

Although we found no studies that were based in Africa, a hospital-based case-control study conducted in Korea (Asia) investigated the effects of interactions between common genetic variants and alcohol consumption on CRC risk. They found that higher levels of alcohol consumption, as calculated based on a standardised definition of a drink (1 drink = 12.5 g of ethanol), were associated with an increased risk of CRC (odds ratio [OR] = 2.47; 95% CI: 1.62–3.76 for heavy drinkers [defined as > 50 g/d] compared to never drinkers; *p* < 0.01).^15^

Austin et al.^16^ reported that the effect of moderate alcohol consumption and the interaction between alcohol and smoking was not well defined and therefore aimed to investigate the relationship between different alcohol consumption levels and colorectal adenomas, and to determine whether smoking modified this relationship.

To go into further detail, eligible patients who underwent a complete colonoscopy were included, with 179 cases and 466 controls. Patients were divided into abstainers; moderate drinkers, defined as > 0 to < 7 drinks/week; and heavy drinkers (≥ 7 drinks/week). The proportion of patients with adenomas was 29.6% among the abstainers, 22.1% among the moderate drinkers and 36.7% among the heavy drinkers.

This study found that the relationship between alcohol consumption and colorectal adenomas varied significantly by smoking history. For individuals who had never smoked, heavy drinkers had increased odds of having an adenoma compared to moderate drinkers (OR = 3.08; 95% CI: 1.50–6.32). No difference was seen for abstainers. Similarly, among individuals who had smoked 1–14 years, heavy drinkers had increased odds of having an adenoma compared to moderate drinkers (OR = 2.61; 95% CI: 1.04–6.51), and no difference was seen for abstainers. Unexpectedly, among individuals who had smoked for 15 or more years, abstainers had increased odds of having an adenoma compared to moderate drinkers (OR = 2.04; 95% CI: 0.91–4.59), while heavy drinkers did not have increased odds of having an adenoma. In conclusion, the consumption of more than seven alcoholic drinks per week did not increase the risk of having a colorectal adenoma and, interestingly enough, moderate alcohol consumption among long-term smokers may potentially decrease the risk of an adenoma compared with abstainers.^17^

Among the worldwide literature, Lindsay et al.^18^ reported that cigarette smoking was associated with CRC even after controlling for screening and multiple risk factors. Chao et al.^19^ reported that long-term smoking was associated with a high risk of CRC, and Prajuli et al.^20^ reported similar findings. A study by Rossi and colleagues^21^ determined that alcohol increased the risk of CRC to a greater extent in men than in women. Petersen et al.^22^ further reported that while alcohol increased the risk of CRC, the type of alcohol was important, and wine seemed to be protective. A study based in China^23^ suggested that alcohol and smoking had an additive effect. Ferdiko and Jenab showed that the amount of alcohol consumed per day was important,^24^ while Ho et al.^25^ and Wang et al.^26^ demonstrated that cessation of alcohol consumption of 10 years or more was associated with a reduction in the risk of CRC. Kotou et al.^27^ showed the association between amount of alcohol and CRC to follow a J-shaped curve and that moderate alcohol consumption may be protective. Walter et al.^28^ found that prediagnostic and heavy alcohol consumption was not only associated with a risk of CRC but also with poorer survival after diagnosis than light drinking; they also found that the protective role of light drinking may be confined to wine. Xu et al.,^29^ in their meta-analysis, found that wine was not associated with the development of CRC in both men and women.

Finally, Ghazaleh et al.^30^ found that alcohol may be associated with an increased risk of CRC in people who carry an *MMR* gene mutation, suggesting a potential role for genetic factors. It is prudent to note that none of the aforementioned studies were done in black African patients.

### Aim and objectives

This study was, thus, designed to investigate the association of smoking and alcohol with CRC, specifically in black Africans. To this end, we firstly sought to determine the relationship between smoking and CRC among black African patients at a single academic centre (objective 1); secondly, we sought to determine the relationship between alcohol and CRC in that single-centre black patient population (objective 2). Our hypothesis underlying this study was that smoking and alcohol significantly predispose individuals of the black African population to CRC.

## Research methods and design

### Study design

This was a hospital-based, case-control study looking at patients attending the Steve Biko Academic hospital GIT clinic.

### Setting

The setting was Steve Biko Academic Hospital, a tertiary institution and one of the main teaching hospitals of the University of Pretoria. More specifically, we looked at subjects who attended the GIT clinic from January 2016 to December 2018.

### Patient selection

Subjects used for the study included black African patients who attended the Steve Biko Academic Hospital GIT clinic between 2016 and 2018. The inclusion criteria were black African patients, age of 18 years or above, who had undergone a colonoscopy for suspected CRC. Furthermore, CRC cases were histology-confirmed and controls were negative. The exclusion criteria were patients who were not of black African descent, below the age of 18 years, and who had not undergone a colonoscopy. Data were collected between June 2019 and March 2020.

### Data measurements

#### Descriptive

The sample was made up of patients of black African descent (belonging or relating to black people from Africa^18^), above 18 years of age, who had undergone a colonoscopy. Family history, indications at the time of colonoscopy (e.g. iron deficiency anaemia, chronic diarrhoea, a mass), sex and comorbid diseases were described in our sample population.

Among our black African patient populations, all of who had undergone a colonoscopy between 2016 and 2018, the exposures observed were smoking and/or had alcohol consumption, with CRC as the outcome. A ‘smoker’ was defined as a person who smoked tobacco in the form of cigarettes, cigars or a pipe prior to the colonoscopy for suspected CRC, irrespective of the outcome. A person who partook of ‘alcohol consumption’ was defined as someone who consumed alcohol prior to the colonoscopy for suspected CRC, irrespective of the outcome. The history of smoking and alcohol was retrospectively obtained from each patient’s physician documentation made at the time the patient was seen in the clinic (i.e. by our viewing of the record files).

Steps to avoid measurement error included the following: use of trained GIT fellows and consultants performing the history-taking and colonoscopies; biopsies being reported by trained anatomical pathology registrars with consultant review of the final results; and data collection being performed by the researcher (eliminating the possibility of untrained data collectors or misunderstandings).

### Statistical considerations

In general, of interest to this study was the association of alcohol use and smoking with CRC.

#### Sample size

When the number of pairs was 68, where each pair, group matched for age, consisted of 1 case and 2 controls, there was a 90.56% power to detect an OR of 3 from a conditional logistic regression. This assumed a probability of exposure of 0.8, and the one-sided test was performed at the 55 significance level. Therefore, a total of at least 68 cases and 136 controls was needed for this study and a total of 110 cases and 220 controls were obtained (Appendix 1).

#### Data analyses

The data collected were recorded in a table format using Microsoft^®^ Excel. Data were analysed using descriptive statistics, such as frequency distribution tables for categorical data and summary statistics for numerical variables. We also determined associations between categorical variables using cross-tabulations and corresponding chi-square tests. The dependent variable was CRC (categorical, binary), and the independent variables included smoking (categorical, binary) and alcohol consumption (categorical, binary).

All statistical analyses were performed using STATA version 14.1. The OR was the measure of the association for the first objective (to determine the relationship between smoking and CRC in patients attending the GIT clinic at the Steve Biko Academic Hospital) and for the second objective (to determine the relationship between alcohol and CRC in patients attending the GIT clinic at the Steve Biko Academic Hospital).

Data analysis employed a logistic regression with the risk factors of smoking and alcohol and the interactions of smoking and alcohol. Particular association was addressed with the margin statement. A *p*-value of ≤ 0.05 was regarded as statistically significant. A knowledgeable biostatistician was consulted throughout the statistical analysis procedure.

### Ethical considerations

Ethical clearance to conduct this study was obtained from the University of Pretoria Faculty of Health Sciences Research Ethics Committee (No. 165/2019) and the management at the Steve Biko Academic Hospital. The study complied with all relevant essential ethical principles.

## Results

**FIGURE 1:**
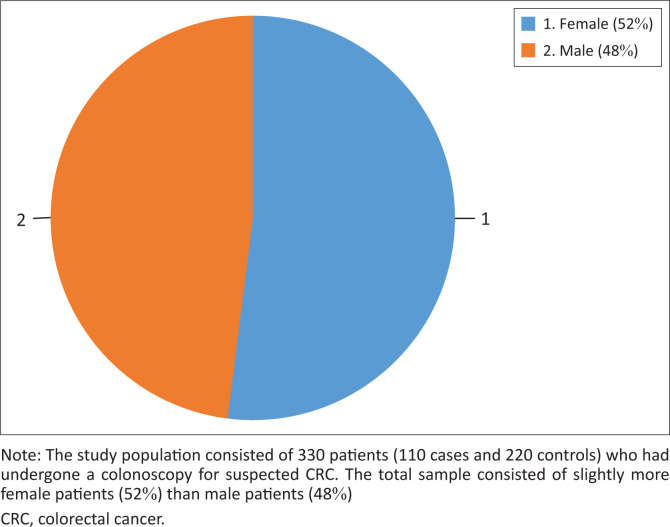
Patient sex.

The final study population was made up of 330 patients (110 cases and 220 controls) who had undergone a colonoscopy for suspected CRC within the time period specified (January 2016 – December 2018). The sex distribution of the patients in the sample is summarised in Figure 1. The sample consisted of slightly more female patients (52%) than male patients (48%).

**FIGURE 2:**
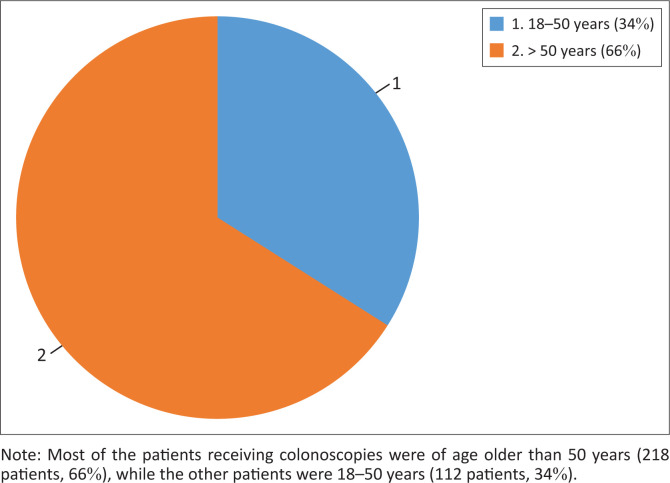
Age breakdown.

Figure 2 summarises the sample’s age breakdown. Results revealed that most of the patients receiving colonoscopies were older than 50 years (218 patients, 66%), while the other patients were 18–50 years (112 patients, 34%).

**FIGURE 3:**
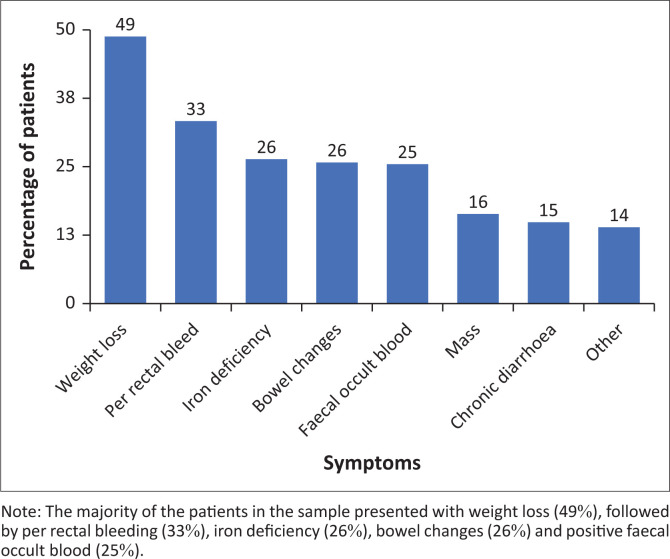
Symptoms.

Close to half of the patients in the sample presented with weight loss (49%), followed by per rectal bleeding (33%), iron deficiency (26%), bowel changes (26%) and positive faecal occult blood (25%) as shown in Figure 3.

**FIGURE 4:**
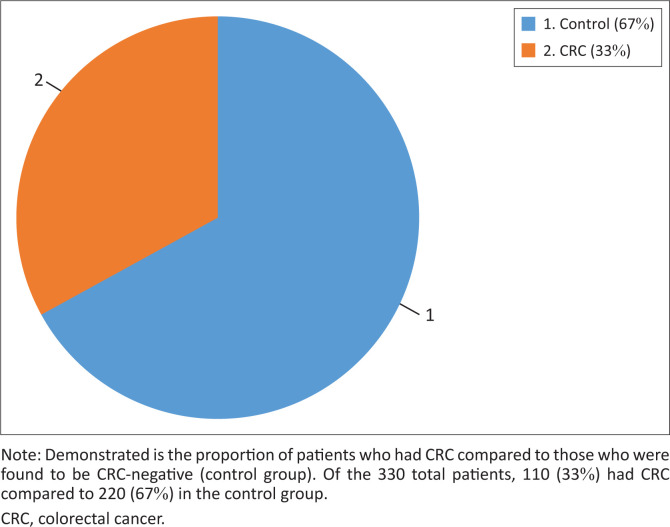
Groups.

Figure 4 shows the proportion of patients who had CRC compared to those who were found to be CRC-negative and were thus in the control group. The results show that of the 330 total patients, 110 (33%) had CRC compared to 220 (67%) in the control group (the latter representing patients who were negative based on histology).

For the 110 patients who had CRC, data on site and stage at presentation are summarised in Table 1. A proportion of 65.5% (72 patients) had CRC in the colon and 34.5% had CRC in the rectum (38 patients). Results revealed that 48.2% of CRC patients were diagnosed at stage I or II, while the other 51.8% were diagnosed at stage III or IV.

**TABLE 1:** Site and stage.

CRC	Option	Frequency (*n*)	Percentage (%)
Site	Colon	72	65.5
	Rectal	38	34.5
Stage	I and II	53	48.2
	III and IV	57	51.8

Note: For the 110 patients who had CRC, a proportion of 65.5% (72 patients) had CRC in the colon and 34.5% in the rectum (38 patients). A total of 48.2% of CRC patients were diagnosed at stage I or II and 51.8% were diagnosed at stage III or IV.

CRC, colorectal cancer

### Is there an association between demographic information and colorectal cancer in patients?

To assess this question, a cross-tabulation, on whether a patient was in the control group had CRC and what their data of associated demographics were, was conducted, and Pearson’s chi-square values were computed as demonstrated in Table 2.

**TABLE 2:** Cross-tabulation of demographics and patient group.

Variable	Option	Group				Total		Odds ratio	*P*
		Control		CRC					
		*n*	%	*n*	%	*n*	%		
Age (years)	18–50	93	42.3	19	17.3	112	33.9	0.29	0.000
	> 50	127	57.7	91	82.7	218	66.1	3.51	
Sex	Female	136	61.8	35	31.8	171	51.8	0.29	0.000
	Male	84	38.2	75	68.2	159	48.2	3.47	
Family history	Yes	4	1.8	17	15.5	21	6.4	9.87	0.000
	No	216	98.2	93	84.5	309	93.6	0.10	
Smoke	Yes	69	31.4	67	60.9	136	41.2	3.41	0.000
	No	151	68.6	43	39.1	194	58.8	0.29	
Alcohol	Yes	92	41.8	19	17.3	111	33.6	0.29	0.000
	No	128	58.2	91	82.7	219	66.4	3.45	

Note: Table showing whether there was an association between demographic information and CRC in patients using Pearson’s chi-square values.

CRC, colorectal cancer.

Results revealed that there was an association between age and CRC, with 82.7% of older patients (> 50 years) presenting with CRC compared to 57.7% of males among the control group. The association was deemed statistically significant according to the Pearson’s chi-square *p*-value of less than 0.001. The OR showed that patients > 50 years are 3.51 times more likely to have CRC than patients in the 18–50 years age group.

Notably, sex was also associated with CRC, with male patients being 3.469 times more likely to have CRC than females (OR = 3.469, *p* < 0.001). Patients with a family history of CRC were found to be 9.87 times more likely to have CRC than those without a family history; thus, family history appeared to increase the chances of being diagnosed with CRC (*p* < 0.001).

Smoking was found to be a risk factor, with smokers being 3.41 times more likely to be diagnosed with CRC (*p* < 0.001). On the other hand, consuming alcohol was surprisingly found to be protective (OR = 0.29, *p* < 0.001). Those who did not consume alcohol were 3.45 times more likely to be diagnosed with CRC than those who do consume alcohol; the result was deemed statistically significant, as the *p*-value was less than 0.05.

### Is there an association between symptoms and colorectal cancer?

To assess this question, cross-tabulations of symptoms and the patient group were conducted. Pearson’s chi-square values were computed, and the results are detailed as follows.

The results (demonstrated in Table 3) indicated that patients who present with chronic diarrhoea were less likely to have CRC, as it was noted that patients who do not present with chronic diarrhoea were 6.80 times more likely to have CRC than those with chronic diarrhoea (*p* < 0.001).

**TABLE 3:** Cross-tabulation of symptoms and patient group.

Variable	Option	Group				Total		Odds ratio	*P*
		Control		CRC					
		*n*	%	*n*	%	*n*	%		
Chronic diarrhoea	Yes	45	20.5	4	3.6	49	14.8	0.15	0.000
	No	175	79.5	106	96.4	281	85.2	6.80	
Bowel changes	Yes	20	9.1	65	59.1	85	25.8	14.44	0.000
	No	200	90.9	45	40.9	245	74.2	0.07	
Mass	Yes	8	3.6	46	41.8	54	16.4	19.05	0.000
	No	212	96.4	64	58.2	276	83.6	0.05	
Weight loss	Yes	92	41.8	69	62.7	161	48.8	2.33	0.000
	No	128	58.2	41	37.3	169	51.2	0.43	
Per rectal bleed	Yes	73	33.2	37	33.6	110	33.3	1.02	0.934
	No	147	66.8	73	66.4	220	66.7	0.98	
Faecal occult blood	Yes	58	26.4	26	23.6	84	25.5	0.87	0.592
	No	162	73.6	84	76.4	246	74.5	1.16	
Iron deficiency	Yes	55	25.0	32	29.1	87	26.4	1.23	0.427
	No	165	75.0	78	70.9	243	73.6	0.81	

Note: Table showing whether there was an association between presenting symptoms and the likelihood of having CRC using Pearson’s Chi-square values.

CRC, colorectal cancer.

Patients with bowel changes were 14.44 times more likely to have CRC than those without bowel changes (*p* < 0.001), and those who presented with a mass were 19.05 times more likely to have CRC than those without a mass (*p* < 0.001). Furthermore, patients who had a clinical complaint of weight loss were 2.33 times more likely to have CRC than those without weight loss (*p* < 0.001).

There was no statistically significant association between CRC and per rectal bleed (*p* = 0.934), faecal occult blood (*p* = 0.592) or iron deficiency (*p* = 0.427).

### Multivariate analysis

A multivariate analysis was performed to see if there was a relationship between smoking and/or alcohol and CRC in the patients obtained for the study. A logistic regression model was fitted with all possible risk factors, adjusting for the confounders of age, sex and family history. Sex was then eliminated because it was not significant. The results are discussed as follows and presented in Table 4.

**TABLE 4:** Impact of alcohol and smoking on predicting colorectal cancer.

Variable	Group	Odds ratio	s.e.	*z*	*P* >|*z*|	95% CI
Smoking	Yes	1.80	0.53	1.97	0.049	1.00–3.21
Alcohol	Yes	0.21	0.09	−3.58	0.000	0.09–0.50
Smoking + alcohol	Yes†	5.93	4.00	2.63	0.008	1.58–22.28
Age	> 50 years	3.74	1.20	4.12	0.000	2.00–7.09
Family history	Yes	12.46	8.35	3.76	0.000	3.35–46.32
Constant	-	0.17	0.06	−5.21	0.000	0.09–0.33

Note: A multivariate analysis demonstrating that smoking (OR = 1.80, *p* = 0.049), age of > 50 years (OR = 3.74, *p* < 0.001), family history (OR = 12.46, *p* < 0.001) and both smoking and consuming alcohol (OR = 5.93, *p* = 0.008) were significant risk factors for CRC. Alcohol appeared to be protective (OR = 0.21, *p* < 0.001).

CI, confidence interval; s.e., standard error.

† , Conditional logistic regression for case-control design (binary variable).

Results showed that smoking (OR = 1.80, *p* = 0.049, CI = 1.00–3.21), age of > 50 years (OR = 3.74, *p* < 0.001, CI = 2.00–7.09), family history (OR = 12.46, *p* < 0.001, CI = 3.35–46.32) and both smoking and consuming alcohol (OR = 5.93, *p* = 0.008, CI = 1.58–22.28) were significant risk factors. Alcohol alone, on the other hand, appeared to be protective (OR = 0.21, *p* < 0.001, CI = 0.09–0.50) according to the *p*-value being less than 0.05.

**FIGURE 5:**
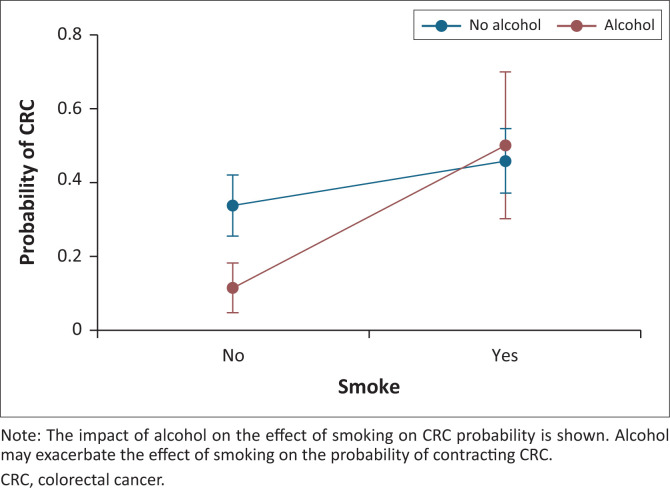
Predicted probability of colorectal cancer with 95% confidence interval.

The impact of alcohol on the effect of smoking on CRC probability is summarised in Figure 5. Alcohol may exacerbate the effect of smoking on the probability of contracting CRC, as shown by the greater steepness of the line compared to the line depicting the difference between smoking and not smoking for those who consume alcohol.

## Discussion

This study aimed to investigate the association of smoking and alcohol with CRC in black Africans. The objective was to determine whether there was a relationship between smoking and/or alcohol and CRC in black patients who attended the GIT clinic at the Steve Biko Academic Hospital. It was hypothesised that both smoking and alcohol are significantly associated with CRC in the black African population. This study revealed that, as per our hypothesis, smoking alone, as well as smoking combined with alcohol, are associated with increased CRC development in black African patients. Interestingly, alcohol used alone had a protective association which proved this part of our hypothesis wrong.

Botteri et al.^10^ found, through their literature review, that the pooled risk estimate (forever vs. never smokers) was 1.25 (95% CI: 1.14–1.37) and that smoking was associated with an absolute risk increase of 6.0 deaths per 100 000 person-years (95% CI: 4.20–7.60). They also noted that in terms of mortality, the association was stronger for cancer of the rectum than that of the colon. However, the study took different population groups into account and not solely black Africans. Our study showed that smoking had a significant association with CRC development. We did not, however, explore the association of smoking on the development of CRC at specific tissue locations, such as the rectum or colon. However, of the patients who had CRC in our study, 65.5% had colonic CRC and 34.5% had rectal CRC.

In our search of the literature, no studies that specifically reported on investigations of the association of alcohol and CRC in Africa were found. In our subsequent clinical data study, alcohol was found to have a protective association. However, we acknowledge that the quantity of alcohol consumption was not taken into account. We also noted that the combination of smoking and alcohol was also significantly associated with CRC. Again, the quantity of alcohol and cigarettes smoked was not taken into consideration, which highlights questions for future areas of research to investigate.

Nearly one-half (48.2%) of the CRC patients in our study population were diagnosed at stages I and II, while a little over one-half (51.8%) were diagnosed at either stage III or IV. As found in the literature review, low socioeconomic status has been shown to be associated with a later stage at diagnosis, bringing about lower resection rates because of advanced disease.^9^ This was similar to what was found in Durban, with a resection rate of 81% for the private sector resembling that of 79% – 89% reported worldwide, while that in the state sector was 61%, reflecting a tendency to present with advanced disease.^9^

As noted in our literature review, most cases are diagnosed after the onset of symptoms. The most common clinical symptoms that were presented in the previous study performed at the Komfo Anokye Teaching Hospital, Ghana, included weight loss (44.80%), bleeding per rectum (39.82%) and abdominal pain (38.91%).^6^ In our study, we found that patients presenting with bowel changes, a mass or weight loss were significantly more likely to have CRC compared to those without symptom complaints. There was no significant association between CRC and per rectal bleed, faecal occult blood, and iron deficiency.

Notably, our study had more female subjects than males. It is important to remark that females are known to practise earlier health-seeking behaviours, as noted by Agyemang-Yeboah et al.^6^ The results also revealed that most of the patients who received colonoscopies were older than 50 years, while the other patients were 18–50 years. This finding was similar to the report by Agtemang-Yeboah et al.^6^

A main limitation of our study was obtaining cases from a single centre only. However, the aims and objectives of the study were met, and the number of cases and controls surpassed the minimal requirements calculated in the statistical analysis. The potential confounders that were identified included the presence of comorbid diseases and their impact on CRC development. In addition, as mentioned earlier, the amount of alcohol and cigarettes consumed differed from patient to patient and was not noted, which may have impacted the outcome. It is also important to keep in mind that how the questions of interest (i.e. smoking or drinking) were posed to the patients is an unknown; the veracity of history-taking and the available history may have been confounding factors as well. Variables such as level of education, background residence (rural vs. urban) and highest level of education were not accounted for because of limited history-taking from retrospective notes and will need further studies. Recall bias (the increased likelihood that subjects with the outcome of interest, CRC, might recall and report exposures compared to those without the outcome) may have also occurred. Language barriers, misinterpretation of the question or poor recording of the answers may also have been issues. Future prospective studies conducted via a structured interview process may help to lessen errors from misinterpretation or the poor recording of important data.

Of note, we do acknowledge that because this was a retrospective case-control study, the findings found can only be used to establish correlation between exposure and outcome, but cannot establish causation. Finally, the single centre used to obtain controls was a tertiary hospital which may not be a true representation of the general population controls (i.e. the proportion of patients without the disease), thereby introducing potential selection bias.

Ideally, it would have been more representative, had one enrolled the entire population (patients presenting at all levels of care) and extracted data on exposure and outcome, thus obtaining the true measure of association. However, because of resource constraints in our population, GIT specialists, fellows, pathologists and the equipment needed for diagnosis are available in our tertiary institution only, with all levels of healthcare ultimately referring to our centre.

In light of the previously mentioned limitations, future studies will benefit from quantifying – as accurately as possible – the quantity and duration of the exposures (in other words, how strong the exposure is). We also acknowledge that individuals used as ‘hospital controls’ tend to be sicker and may have worse habits than those among the general population. Further studies should, therefore, be performed at more local or district hospital levels.

Furthermore, we acknowledge that sampling at one place in Africa and thereafter making claims for all black Africans would be somewhat misleading. However, it is our hope that identifying the role of these exposures in this subset of individuals may lead to larger trials which better represent this group of individuals, as current clinical practice is based on extrapolating what has already been established as causes of CRC in other population groups. Finally, the significance of smoking as well as smoking combined with alcohol found in this study shed light on associated factors that one may try to target through behavioural modification techniques and patient education. We anticipate that our findings will contribute to the overall knowledge towards helping to alleviate CRC disease burden in communities through evidence-based education.

## Conclusion

### Research background

There are currently no studies reported that have investigated the association of smoking and alcohol with the development of CRC in black Africans.

### Research motivation

The prevalence of CRC in black Africans is increasing and showing an involvement of younger patients. It is therefore important to identify all of the possible associations that may be contributing to this increase.

### Research objectives

The main objective of this study was to assess the effect that smoking and alcohol may have on CRC development in black Africans. This study revealed that smoking alone and in combination with alcohol are associated with increased CRC development.

### Research methods

This was a retrospective case-control study focused on the histological outcomes of patients exposed to smoking and/or alcohol. This study is the first of its kind in Africa.

### Research results

This study suggests that smoking alone or in combination with alcohol is associated with CRC, while alcohol alone has a protective effect. The limitations of this study are that it is from a single, tertiary centre and that neither alcohol nor smoking was quantified.

### Research conclusions

Smoking in combination with alcohol was found to be associated with patients who developed CRC. The surprising finding is that alcohol alone may have a protective association. Smoking, as in most population groups, is a risk factor for CRC. The observed protective role of alcohol needs to be confirmed in larger studies. Alcohol in moderation has been found to be protective in other systems, such as the cardiovascular system.

### Research perspectives

Smoking is associated with CRC in black Africans. Similarly, alcohol, when combined with smoking, is associated with the development of CRC, but has a protective association when used alone.

## Appendix 1

**TABLE 1-A1:** Statistical calculation.

Parameters	Values
Test significance level (*α)*	0.050
1 or 2 sided test?	1.000
Probability of exposure, P(E)	0.800
Exposure vs. covariates, R^2^	0.000
Number of cases per pair, M_i_	1.000
Number of controls per pair, M_2_	2.000
Odds ratio, (OR)	3.000
Power (%)	90.056
Number of pairs (*N)*	68.000
